# *Artocarpus hirsutus Lam* Leaf Extract-Evaluation of Analgesic and Anti-Inflammatory Activity

**DOI:** 10.1155/2022/4179487

**Published:** 2022-11-26

**Authors:** M. Karthik, Ullas Prakash D'Souza, Prasanna Shama Khandige, Vandana Sadananda, S. Gowrish, Savadamoorthi Kamatchi Subramani

**Affiliations:** ^1^Nitte (Deemed to be University), NGSM Institute of Pharmaceutical Sciences (NGSMIPS), Department of Pharmacology, Mangaluru, India; ^2^Nitte (Deemed to be University), AB Shetty Memorial Institute of Dental Sciences (ABSMIDS), Department of Conservative Dentistry and Endodontics, Mangaluru, India; ^3^Jazan University, College of Dentistry, Department of Restorative Dental Sciences, Division of Operative Dentistry, Jazan, Saudi Arabia

## Abstract

The study involved extraction, identification, and evaluation of pharmacological activities of the phytochemicals present. *Artocarpus hirsutus Lam*, commonly known as Wild jack is a greatly valued medicinal plant, which belongs to the plant family Moraceae. Preliminary phytochemical screening studies indicated the presence of flavonoids, saponins, tannins, glycosides, and alkaloids. This study estimated the analgesic and anti-inflammatory prospective of ethanolic leaf extract of *Artocarpus hirsutus Lam*. The findings showed that at various doses of 100, 200, and 400 mg/kg body weight when administered orally to rats, analgesic effects were produced, also the anti-inflammatory effect studied by carrageenan-induced rat paw edema test showed major anti-inflammatory action. The result indicates that the leaf extract of *Artocarpus hirsutus Lam* possesses major analgesic and anti-inflammatory activity and therefore requires further investigations to better understand the mechanism of action.

## 1. Introduction

Inflammation and pain are manifestations of many diseases including oral diseases. Commonly used drugs in such scenarios include nonsteroidal anti-inflammatory drugs (NSAIDs) and opiates. However adverse reactions are observed on the administration of these drugs such as renal damage, respiratory depression, gastrointestinal disturbances, renal damage, and including dependence [1–10]. This has led the thought process of the researchers towards natural plants and ayurvedic medicine science which would serve as novel anti-inflammatory and analgesic drugs with possibly exhibition of fewer side effects [1–20].

*Artocarpus hirsutus lam* is a tropical evergreen tree belonging to the genus Artocarpus and Moraceae family extensively known for its edible fruit and wood. *Artocarpus hirsutus Lam* is native to India, primarily in South India. *Artocarpus hirsutus Lam* is an evergreen canopy tree that can grow to a height of 35 m and about 4.5 m in girth. The various morphological parts of the plant are effective against skin diseases, intrinsic haemorrhage, and poisons; bark infusion is used to cure small pimples and cracks present on the skin. The powdered bark is used to treat sores, dried leaves are applied to buboes and hydrocele [21–67].

The present research work aimed to identify and isolate the phytoconstituents from the ethanolic leaf extract of *Artocarpus hirsutus Lam* and evaluate the analgesic and anti-inflammatory activities.

## 2. Methodology

### 2.1. Plant Leaf Collection, Authentication and Extraction

The leaves of the plant *Artocarpus hirsutus Lam* collected from Deralakatte, Mangaluru, Karnataka were authenticated by a botanist. The fresh leaves were sun dried and subsequently pulverized using a mechanical grinder. The dry powder was macerated in ethanol for a duration of 7 days with occasional stirring. The total extracts of the leaves were distilled, filtered, concentrated using a rotary evaporator, and stored in a desiccator.

### 2.2. Preliminary Qualitative Phytochemical Investigations

Presence of diverse phytochemicals in the total extracts of the leaves of *Artocarpus hirsutus Lam* was assessed by qualitative test for preliminary phytoconstituents.

#### 2.2.1. Test for Alkaloids

*(1) Dragendrof f's Test*. In 5 mL of distilled water, 0.5 g of ethanolic leaf extract was dissolved, to which 2 M hydrochloric acid was added for the reaction to initiate. Subsequently, Dragendroff's reagent (1 mL) was added to the above solution. An orange red precipitate indicates the presence of alkaloids.

*(2) Hager's Test*. Hager's reagent was added to 1 mL of ethanolic extract of leaves. Yellowish colored precipitate formed confirms the presence of alkaloids.

*(3) Wagner's Test*. 1.5 mL of hydrochloric acid was added to 1 mL of ethanolic extract of the leaf. Subsequently few drops of Wagner's reagent were added. Buff colored precipitate formation indicates the presence of alkaloids.

*(4) Mayer's Test*. Mayer's reagent was added to 1 mL of ethanolic extract. The presence of alkaloids was confirmed by observing the formation of pale yellow or white precipitate.

#### 2.2.2. Test for Reducing Sugar

*(1) Molisch Test*. Freshly prepared 20% alcoholic *α*-naphthol was added to 2 mL of the leaf extract to which 2 mL of concentrated sulphuric acid was added dropwise along the walls of the test tube to facilitate the formation of a layer and to avoid mixing. The formation of the purple ring at the layer formed is an indication for the presence of carbohydrates which vanishes on the addition of an excess of an alkaline reagent.

*(2) Benedict's Test*. Benedict's solution was added to the leaf ethanolic extract and heated. The formation of a brick red color confirms the presence of carbohydrates.

*(3) Fehling's Test*. 10 mg of the ethanolic leaf extract was dissolved in 1 mL of water. 1 mL of Fehling's A and Fehling's B solution were added. The presence of reducing sugar was confirmed by observing the formation of brick red color.

*(4) Tollen's Test*. In 1 mL of water 10 g of ethanolic leaf extract was dissolved. 1 mL of Tollen's solution was added and heated on a water bath. The formation of a black precipitate or black silver mirror along the sides of the test tube confirms the existence of reducing sugars.

#### 2.2.3. Test for Flavonoids

*(1) Shinoda Test*. 0.5 mL of ethanolic leaf extract was heated, and subsequently hydrochloric acid (10 drops) and magnesium powder were added. The color change of the solution to reddish brown proves the presence of flavonoids.

#### 2.2.4. Test for Saponins

10 mg of ethanolic extract was dissolved in water and shaken vigorously. The appearance of foam proves the presence of saponins.

#### 2.2.5. Test for Tannins

To 0.5 g of ethanolic leaf extract, 5 mL of chloroform was added. A reddish pink color formed by the addition of 1 mL of acetic anhydride and 2 drops of sulphuric acid to the solution confirms the presence of tannins.

#### 2.2.6. Tests for Steroids

*(1) Liebermann-Burchard's Test*. 10 mg of the ethanolic extract was dissolved in acetic anhydride. The solution was heated and cooled. Concentrated sulphuric acid (1 mL) was incorporated slowly, along the side of the test tube. The development of a greenish color confirms the presence of steroids.

*(2) Salkowski Reaction*. To the ethanolic leaf extract, 1 mL of concentrated sulphuric acid was added along the side of a test tube. The mixture was allowed to stand. The red color formed in the chloroform layer confirms the presence of steroids.

#### 2.2.7. Test for Triterpenoids

The leaf extract was dissolved in acetic anhydride. The solution was heated and allowed to cool. Concentrated sulphuric acid (1 mL) was added along the sides of the test tube. Violet color formation confirms the presence of triterpenoids.

#### 2.2.8. Test for Glycosides

*(1) Molisch Test*. 2 mg of ethanolic leaf extract was dissolved in 10 mL of water which was shaken and filtered. To the concentrated filtrate 2 to 3 drops of Molisch reagent was added, mixed, and subsequently concentrated sulphuric acid (2 mL) was added along the sides of the test tube. Reddish-violet ring proved the presence of glycosides.

#### 2.2.9. Test for Resins

A few drops of acetone were added to 1 mL ethanolic extract and dissolved. This solution was added to distilled water. Turbidity confirms the presence of resins.

### 2.3. Pharmacological Investigations

#### 2.3.1. Selection of Animals

Male albino rats, Wistar strain of 4–6 weeks weighing (150–200 g), albino mice (25–30 g) were obtained from central animal house NUCARE, Derlakatte, Mangaluru for the study after obtaining approval from the Institutional Ethics Committee for animal experimentation. Animals were grouped and housed in cages and maintained under basic laboratory conditions (temperature 20–25°C) with dark and light cycle (12 h/12 h). They were fed a basic dry pellet diet and water ad libitum. All investigations and experiments were conducted based on the guidelines of the Committee for the Intent of Control and Supervision of Experiments on Animals (CPCSEA), New Delhi, India and was affirmed by the Institutional Animal Ethics Committee (IAEC) of NGSM Institute of Pharmaceutical Sciences–NGSMIPS/IACE/MAY-2017/60.

#### 2.3.2. Acute Toxicity Studies

Preliminary pharmacological studies were carried out to evaluate the acute toxicity and LD50 of the ethanolic extract of the leaves of *Artocarpus hirsutus* [52, 53]. The acute toxicity studies were conducted in adult male albino rats which were weighing about 150–200g, by the “up and down” method (OECD guidelines 425). Overnight fasted animals were given ethanolic leaves extract at a dose of 2000 mg/kg body weight orally. The animals were noticed for the next 4 hours for the general behavioral changes, neurological profile, and autonomic profiles, and last for death after 24 hours. The result attained was tabulated in Irwin's table.

The following check list are selected for this:

(i) Behavioral profile
    Awareness-alertness, passivity, stereotype, visual placing
    Mood-fearfulness, grooming, irritability, restlessness
(ii) Neurological profile
    Motor activity-corneal reflex, gait, grip, pain response, reactivity, startle response, spontaneous response, touch response, and tremor
(iii) Autonomic profile
    Defecation, heart rate, pilo erection, respiratory rate, urination, and writhing

#### 2.3.3. Dose Selection

For conducting analgesic and anti-inflammatory activity, three dose levels were selected [53]. The middle dose was approximately 1/10th of the maximum dose which was administered while evaluating acute toxicity (i.e., 1/10th of 2000 mg/kg body weight–200 mg/kg body weight), and the high dose was twice that of 1/10th dose (i.e., 400 mg/kg body weight) and a low dose which was of 50% of 1/10th dose (i.e., 100 mg/kg body weight) was administered. The dried crude ethanolic leaves extract of *Artocarpus hirsutus Lam* was suspended in 2% w/v of Gum acacia in normal saline for administration orally.

#### 2.3.4. Assessment of *In Vitro*Anti-Inflammatory Activity Inhibition of Albumin Denaturation

The reaction mixture (5 mL) containing 0.2 mL of egg albumin, phosphate buffer saline 2.8 mL with pH 6.4, and 2 mL of ethanolic leaves extract of different concentration was taken with a final concentration of 31.25, 62.5, 125, 250, 500, and 1000 *μ*g/mL [54–67]. Distilled water was taken as blank/control which had a similar volume as that of the reaction mixture. Both these mixtures were incubated at 37 ± 2°C in an incubator for 15 min and then heated for 5 min at a temperature of 70°C. After cooling, absorbance was measured at 660 nm. Diclofenac sodium as a reference at concentrations 78.125, 156.25, 312.5, 625, 1250, and 2500 *μ*g/mL was treated similarly, and absorbance was measured.

*(1) Evaluation*. The turbidity formed was measured spectrophotometrically at 660 nm. The calculation for percentage inhibition of protein denaturation was done by the following formula:

(1) $$\begin{array}{c} \%\;inhibition=\left[1-\frac{v_{t}}{v_{c}}\right]\times 100\text{,} \end{array}$$

where *V*_*t*_ = absorbance of test sample, *V*_*c*_ = absorbance of control.

#### 2.3.5. *In Vivo* Methods to Detect Analgesic and Anti-Inflammatory Activity

*(1) Analgesic Activity*. Tail immersion method.

  Group I: control
  Group II: standard, Pentazicine (10 mg/kg)
  Group III: test drug (100 mg/kg)
  Group IV: test drug (200 mg/kg)
  Group V: test drug (400 mg/kg)

The tails of the albino rats (Table 1) were gently immersed in a thermostatic organ bath with a temperature maintained at 55 ± 10°C. The animals which withdrew the tail from hot water within 3–5 seconds were selected for the test. The reaction time of all animals towards thermal heat was noted. Group III, Group IV, and Group V animals were administered ethanolic extract in 2% w/v Gum acacia in normal saline orally. Group II was given the standard drug Pentazocine (10 mg/kg) intraperitoneally and Group I received 2% w/v of Gum acacia (2 mL/kg) with normal saline.

*(2) Evaluation*. Animals from all the groups were exposed to hot water subsequent to the administration of the test and reference compounds. Reaction time was noted at 30 and 90 mins interval, also the reaction time was noted as and when the animals withdrew their tail completely from the hot water bath. The mean reaction time in each group was evaluated statistically.

(2) $$\begin{array}{c} \%\;increase\;in\;reaction\;time=\left[\frac{{RT}_{AT}}{{RT}_{BT}}-1\right]\times 100\text{.} \end{array}$$

RT_AT_ and RT_BT_ are the reaction time after treatment and before treatment, respectively.

Acetic acid induced writhing method [56].

  Group I: control.
  Group II: standard, Diclofenac sodium (10 mg/kg)
  Group III: test drug (100 mg/kg)
  Group IV: test drug (200 mg/kg)
  Group V: test drug (400 mg/kg)

The first group of animals were administered acetic acid 06% v/v. i.p., which were considered as control. The second group was administered the standard drug Diclofenac sodium (10 mg/kg body weight, i.p). The third, fourth and fifth group animals were administered 100, 200, and 400 mg/kg body weight ethanolic extract which was suspended with 2% gum acacia 30 min prior (Table 2).

*(3) Evaluation*. The writhing action was indicated by the stretching of the abdomen along with the simultaneous stretching of at least one hind limb. This action was observed for 30 min. Percentage inhibition calculation was carried out by using the following formula:

(3) $$\begin{array}{c} percentage\;inhibition=\left[1-\frac{R_{t}}{T_{c}}\right]\times 100\text{.} \end{array}$$

*R*_*t*_ = Mean number of writhes in treated groups, *R*_*c*_ = Mean number of writhes in control groups.

Anti-inflammatory screening method Carrageenan induced rat paw edema method [57].

  Group I: control
  Group II: standard, Diclofenac sodium (10 mg/kg)
  Group III: test drug (100 mg/kg)
  Group IV: test drug (200 mg/kg)
  Group V: test drug (400 mg/kg)

Albino rats weighing between 150–200 g were taken for the test. Prior to the day of the experiment the animals were starved overnight with water ad libitum. An identification mark was made on both the hind paws just below the tibio-tarsal junction. Each time the paw was dipped up to the mark into the mercury column of the plethysmograph to get constant paw volume. The second group was administered a standard drug, Diclofenac sodium 10 mg/kg body weight. The third, fourth and fifth groups received ethanolic leaves extract at 100, 200, and 400, respectively. Acute inflammation was induced after thirty minutes of administration of the drug by injecting into the subplantar region of the left hind paw 0.1 mL of 1% carrageenan (Table 3).

*(4) Evaluation*. The paw volume was measured with the use of a plethysmograph at 0, 0.5, 1, 2, and 3 hours subsequent to the carrageenan injection. The difference between 0 hour and consequent readings was considered as edema volume. The calculation for percentage inhibition of edema was carried out by using the following formula:

(4) $$\begin{array}{c} \%\;edema\;inhibition=\left[1-\frac{V_{t}}{V_{c}}\right]\times 100\text{.} \end{array}$$

*V*_*t*_ = volume of paw edema in drug treated group *V*_*c*_ = volume of paw edema in control group.

*(5) Statistical Analysis*. The data obtained were evaluated using one way analysis of variance (ANOVA), followed by posthoc test (Scheffe's *T*-Test) using graph pad SPSS software version 16.0 where *p* value less than 0.05 was taken as statistically significant.

## 3. Results

### 3.1. Preliminary Phytochemical Analysis of Ethanolic Extract of *Artocarpus hirsutus Lam*

Phytochemical analysis results Table 4).

### 3.2. Acute Toxicity Studies

The ethanolic extract of leaves of *Artocarpus hirsutus Lam* up to 2000 mg/kg body weight by the oral route of administration were found to be safe. Even after 24 hours the animals were found to be well tolerated. There was no indication of toxicity and death. Three doses, 100 mg/kg, 200 mg/kg, and 400 mg/kg body weight were selected for the experiment.

### 3.3. Analgesic Activity

Effect of ethanolic extract of Artocarpus hirsutus Lam on tail immersion method Tables 5 and 6).

### 3.4. Anti-Inflammatory Activity

Effect of ethanolic extract of Artocarpus hirsutus Lam on carrageenan induced rat paw edema method Table 7).

## 4. Discussion

The present study was designed to evaluate the analgesic and anti-inflammatory activity of ethanolic leaf extract of *Artocarpus hirsutus Lam*. The peripheral analgesic activity was carried out using the chemical induced writhing method (Table 6) and central analgesic activity by tail flick using immersion of the tail (Table 5). The carrageenan induced paw edema model was carried out to identify the acute inflammatory activity.

Preliminary phytochemical screening of ethanolic leaves extract of *Artocarpus hirsutus Lam* signifies the presence of saponins, tannins, flavonoids, triterpenoids, glycosides, alkaloids, and resins (Table 4). When the ethanolic extract of the leaves were administered orally at the doses of 200 and 400 mg/kg, significant analgesic activity was exhibited (*p* < 0.05) by inhibition of writhing induced by acetic acid in the mice. The writhing is associated to the increase in levels of PGE2 and PGF2 in the peritoneal fluid and elevation in levels of lipoxygenase products by the peritoneal injection of acetic acid [60]. Acetic acid induced writhing methods also release endogenous substances like bradykinin, histamine, and serotonin, which activates the sensory nerve endings [61]. Antinociceptive action of ethanolic extract of *Artocarpus hirsutus Lam* leaves could be due to its action on visceral receptors which are reactive to acetic acid thereby preventing prostaglandin synthesis.

In the tail immersion test the pain produced by the thermal stimulus is characteristic for central-mediated activity [62]. Opioid agents like morphine bring about analgesic action via supra spinal and spinal receptors [63]. This activity could be due to the stimulation of the periaqueductal gray matter area to discharge endogenous peptides [64]. These endogenous peptides crash the spinal cord and inhibit pain impulse transmission at the synapse in the dorsal horn. The possible actions of ethanolic extract of *Artocarpus hirsutus Lam* leaves may be due to its action on the central receptors or promoted discharge of endogenous opioid peptides. In this study, the action of the drug extract at a dose of 100 mg/kg was not significant. But it showed a significant increase (*p* < 0.05) at a dose of 200 mg/kg body weight and tail flick latency was highest (*p* < 0.05) at a dose of 400 mg/kg body weight at 90 min. Hence an expressive increase in reaction time and decrease in writhing are generally taken as significant parameters of analgesic activity in the heat conduction method and acetic acid induced writhing test respectively.

The carrageenan technique was chosen due to its sensitivity in identifying anti-inflammatory agents which are orally active, mainly in the acute stage of inflammation. Carrageenan is used to produce acute inflammation and is biphasic. The initial phase (0–2.5 hours after carrageenan injection) mainly occurs due to the concordant release of mediators such histamine, kinins, and serotonin. The next phase is related with an increase in the production of prostaglandin, oxygen derived free radicals, and inducible cyclooxygenase production [65, 66]. The carrageenan induced paw edema method in rats is avowed to be reactive to cyclooxygenase (COX) inhibitors as well as lipoxygenase inhibitors thereby restricting the enzyme COX involved in the synthesis of prostaglandins. When compared to the control, the dose of 400 mg/kg body weight at 30, 60, 120, and 180 minutes showed a significant inhibition (*p* < 0.05) of rat paw edema induced by carrageenan, respectively (Table 7). Based on the result it can be concluded that the inhibitory action of ethanolic leaves extract on Carrageenan induced inflammation in rat paw may be because of the restriction of the enzyme cyclooxygenase which further leads to restriction in the synthesis of prostaglandin (Tables 8 and 9).

The denaturation of protein method was selected for the evaluation of in vitro anti-inflammatory activity of the ethanolic extract of *Artocarpus hirsutus Lam*. Tissue protein denaturation is the main causes of inflammatory and arthritic disease. Substances that restrict the denaturation of protein are meritorious in the anti-inflammatory drug development [67]. From the results, it can be deduced that the ethanolic extract of *Artocarpus hirsutus Lam* is more effective in less concentration than that of diclofenac sodium and thus it has anti-inflammatory activity.

## 5. Conclusion

*Artocarpus hirsutus Lam* has been used in folklore, Ayurvedic and traditional medicines for the treatment of numerous health concerns. Through this study, the phytoconstituents present in ethanolic leaf extracts of *Artocarpus hirsutus Lam* were identified. Using animal models, it was concluded to have good analgesic and anti-inflammatory potential. This study should enable researchers to identify and isolate new constituents from *Artocarpus hirsutus Lam*, understand the mechanism of action of the phytoconstituents and formulation of drugs for various diseases.

## Figures and Tables

**Table 1:** Material and method.

Animals	Albino rat
Sex	Male/female
Body weight	150–200 g
Number of groups	5
Number of animals in each group	6
Vehicle	Normal saline
Route of administration	Standard-i.p, test-p.o
Water and food	Ad libitum

**Table 2:** Material and method: chemical induced writhing method.

Animal	Albino mice
Sex	Male/female
Body weight	20–25 g
Number of groups	5
Number of animals in each group	6
Vehicle	Normal saline
Route of administration	Standard-i.p., test-p.o
Water and food	Ad libitum

**Table 3:** Material.

Animal	Albino mice
Sex	Male/female
Body weight	20–25 g
Number of groups	5
Number of animals in each group	6
Vehicle	Normal saline
Route of administration	Standard-i.p, test-p.o
Water and food	Ad libitum

**Table 4:** Phytochemical analysis results.

Test	Result
Alkaloids	
a Dragendroff's test	+ve
b Hager's test	+ve
c Wagner's test	+ve
d Mayer's test	+ve
Carbohydrates	
a Molisch's test	+ve
b Benedict's test	+ve
c Fehlings's test	+ve
Flavonoids	+ve
Saponins	+ve
Tannins	+ve
Steroids -salkowaski test	+ve
Glycosides -Molisch's test	+ve
Resins	−ve

**Table 5:** Effect of ethanolic extract of *Artocarpus hirsutus Lam* on tail immersion method.

Group	Treatment	Dose (mg/kg)	Reaction time in seconds			
			Before treatment	30 min	60 min	90 min
I	Control	2	1.84 ± 0.03	1.82 ± 0.05	2.85 ± 0.05	3.11 ± 0.4
II	Standard	10	2.13 ± 0.12	6.0 ± 0.55^a^	6.5 ± 0.29^a^	9.4 ± 0.04^a^
III	Ethanolic extract	100	1.8 ± 0.05	1.58 ± 0.04	2.2 ± 0.08	3.4 ± 0.09
IV	Ethanolic extract	200	1.81 ± 0.08	3.3 ± 0.3^ab^	3.7 ± 0.39^ab^	4.7 ± 0.59^ab^
V	Ethanolic extract	400	1.9 ± 0.071	4.9 ± 0.088^ab^	5.9 ± 0.076^ab^	6.4 ± 0.099^ab^

*p* < 0.05 is considered as significant, *a* = *p* < 0.05 significant compared to control, *b* = *p* < 0.05 significant compared to standard.

**Table 6:** Effect of ethanolic extract of *Artocarpus hirsutus Lam* by acetic acid induced writhing method.

Group	Treatment	Dose (mg/kg)	Number of writhings
			Mean ± SEM
I	Control	0.1 mL	91 ± 0.36
II	Standard (diclofenac)	10	22.3 ± 0.55^a^
III	Ethanolic extract	100	83 ± 0.85
IV	Ethanolic extract	200	61.6 ± 1.0^ab^
V	Ethanolic extract	400	53.16 ± 0.47^ab^

*p* < 0.05 is considered as significant, *a* = *p* < 0.05 significant compared to control, *b* = *p* < 0.05 significant compared to standard.

**Table 7:** Effect of ethanolic extract of *Artocarpus hirsutus Lam* on carrageenan induced rat paw edema method.

Group	Treatment	Dose (mg/kg)	30 min	60 min	120 min	180 min
I	Control	2 mL/kg	0.55 + 0.04	0.63 ± 0.04	0.64 ± 0.04	0.64 ± 0.03
II	Standard	10 mg/kg	0.17 ± 0.02^a^	0.26 ± 0.04^a^	0.16 ± 0.02^a^	0.16 ± 0.03^a^
III	Ethanolic extract	100 mg/kg	0.52 ± 0.02	0.54 ± 0.06	0.51 ± 0.04	0.47 ± 0.06
IV	Ethanolic extract	200 mg/kg	0.42 ± 0.03^ab^	0.47 ± 0.04^ab^	0.42 ± 0.04^ab^	0.4 ± 0.03^ab^
V	Ethanolic extract	400 mg/kg	0.31 ± 0.08^ab^	0.32 ± 0.05^ab^	0.35 ± 0.07^ab^	0.25 ± 0.09^ab^

*p* < 0.05 is considered as significant, *a* = *p* < 0.05 significant compared to control, *b* = *p* < 0.05 significant compared to standard.

**Table 8:** Absorbance of ethanolic extract of *Artocarpus hirsutusn Lam* leaves at 660 nm.

Concentration	Absorbance	% inhibition
Control	0.04	—
31.25	0.066	65
62.5	0.073	82.5
125	0.079	97.5
250	0.083	107.5
500	0.094	135
1000	0.103	157.5

**Table 9:** Absorbance of standard (diclofenac sodium).

Concentration	Absorbance	% inhibition
Control	0.04	—
78.125	0.025	29
156.25	0.063	57.5
312.5	0.072	80
625	0.110	175
1250	0.122	205
2500	0.13	225

## Data Availability

The data used to support the study are included in the paper.
